# Advances in Isotope Labeling for Solution Nucleic Acid Nuclear Magnetic Resonance Spectroscopy

**DOI:** 10.1002/cplu.202400752

**Published:** 2025-05-02

**Authors:** Stefan Hilber, Solomon Kojo Attionu, Theodore Kwaku Dayie, Christoph Kreutz

**Affiliations:** ^1^ Institute of Organic Chemistry and Center for Molecular Biosciences Innsbruck (CMBI) University of Innsbruck Innrain 80/82 6020 Innsbruck Austria; ^2^ Department of Chemistry and Biochemistry University of Maryland, College Park MD 20782 USA

**Keywords:** RNA nuclear magnetic resonance, segmental labeling, stable isotope labelling

## Abstract

The availability of structural biology methods for nucleic acid still lags behind that of proteins, as evidenced by the smaller number of structures (DNA: 2513, RNA: 1899, nucleic acid–protein complexes: 13 842, protein: 196 887) deposited in the protein database. The skewed ratio of nucleic acid structures, relative to proteins (≈1:50), is inverted with respect to the cellular output of RNA and proteins in higher organisms (≈50:1). While nuclear magnetic resonance (NMR) is an attractive biophysical tool capable of bridging this gap at the molecular level, the conformational flexibility, line broadening, and low chemical shift dispersion of nucleic acids have made the NMR method challenging, especially for structures larger than 35 nucleotides. The incorporation of NMR‐active isotopes is a f strategy to combat these problems. Significant strides made to push the size limits of nucleic acid structures solved by NMR using chemoenzymatic ^13^C‐ methyl and aromatic ^15^N‐ and ^19^F–^13^C‐labeling are reviewed and challenges and opportunities are evaluated. Combining these isotopic labeling patterns with superior NMR spectroscopic properties, and new DNA/RNA synthesis methods (palindrome‐nicking‐dependent amplification and segmental labeling and site‐specific modifications by template‐directed tension), may stimulate advances in NMR studies of large DNA/RNA and their complexes with important biological functions.

## Graphical abstract

Recent advances in the field of nucleic acid nuclear magnetic resonance (NMR) are presented. Stable isotope labeling protocols and new approaches to segmentally label nucleic acid with NMR‐active residues are summarized. The novel methods will have a strong impact on the future of nucleic acid NMR and the characterization of structure and dynamics of large RNAs and DNAs will be possible.

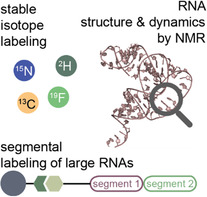

## 1 Introduction

Nuclear magnetic resonance (NMR) spectroscopy is an indispensable tool for characterizing nucleic acid structure and dynamics. Nucleic acids fulfill numerous crucial cellular functions and a deep understanding of their structure and dynamics is needed. Even though the classical structural determination methods of X‐Ray crystallography and NMR spectroscopy still play crucial roles in these endeavors, cryo‐electron microscopy (cryo‐EM) is increasingly becoming very competitive to obtain high‐resolution structures.^[^ ^1^ ^]^ Nonetheless, in addition to obtaining high resolution structures, the interconversion between various conformational states is fundamental to the function of biomolecules.^[^ ^2^ ^]^ For instance, a protein or nucleic acid may require switching between different conformations to transduce specific signals into a biological outcome.^[^ ^3^ ^]^ Such conformational transitions drive folding/unfolding transitions, signalling by binding or releasing a ligand, or mediate enzymatic reactions. NMR remains the method of choice to characterize the folding landscape of a biomolecule on various time scales ranging from picoseconds to seconds,^[^ ^4^ ^]^ but still, some challenges remain in biomolecular solution NMR spectroscopy. The foremost challenge in solution NMR remains increased linewidths as a function of increasing molecular weight. As biomolecules grow in molecular mass, the associated transverse relaxation rate (*R*_2_) increases. This in turn leads to broad signals and a poor signal‐to‐noise (S/N) ratio to work with.^[^ ^5^ ^]^ Such broad lines severely limit the application of several solution NMR experiments, including relaxation dispersion pulse programs;^[^ ^6^ ^]^ the low S/N ratio can prevent the extraction of reliable relaxation parameters. The second crippling challenge for large biomolecules is increase in signal overlap with increase in size. Unlike proteins with 20 amino acid building blocks, nucleic acids feature only four basic nucleotide building blocks—(deoxy)adenosine, (deoxy)cytidine, (deoxy)guanosine, and thymidine/uridine.^[^ ^7^ ^]^ Even worse, these four building blocks utilize the same (deoxy)ribose sugar building block and aromatic nucleobases, which all have similar chemical structures. This translates into a lack of chemical shift dispersion, and thus, extensive signal degeneracy. Therefore, with increasing size and a higher number of C—H/N—H correlations, the probability of resonance overlap increases dramatically for nucleic acids. To meet these challenges, extensive efforts have gone into improving the NMR hardware (e.g., cryoprobe technology),^[^ ^8^ ^]^ improving NMR pulse sequence design,^[^ ^9^ ^]^ and into optimizing the sample preparation of biomolecules.^[^ ^10^ ^]^ All these efforts are aimed at pushing the size limit of solution NMR spectroscopy to go beyond the current 30 nucleotide (nt) median size.^[^ ^11^ ^]^ With respect to sample preparation, ^13^C/^15^N and ^2^H labeling protocols developed for proteins have shown promise for NMR studies of biomolecular protein complexes in the 100–500 kDa size range, if deuteration is combined with selective ^13^C‐methyl labeling.^[^ ^12^ ^]^ Stable isotope (SI) labeling with ^13^C/^15^N‐labeled (deoxy) rNTPs and T7 RNA polymerase in vitro transcription or a DNA polymerase extension from DNA templates is still the most widespread technique to produce SI‐labeled RNAs and DNAs.^[^ ^13^ ^]^ However, solid‐phase nucleic acid synthesis is gaining attraction as a well‐suited method to produce nucleic acids for NMR, allowing facile position‐specific labeling of residues.^[^ ^14^ ^]^ Even though this approach is limited to ≈70 nt, this size limit can be extended with segmental labeling study in large systems. In this approach, parts of the macromolecule can either be made NMR silent or visible by restricting the SI labeling to domains of the nucleic acid using either solid‐phase synthesis or primer‐facilitated enzymatic synthesis. Lukavsky and coworkers introduced this approach for NMR analysis of RNAs with more than 100 nt using enzymatic ligation.^[^ ^15^ ^]^

In this review, we recapitulate recent advances in the SI labeling of nucleic acids with a special focus on DNA ^13^C methyl and RNA aromatic ^19^F‐^13^C‐labeling, which unlocks the very powerful transverse relaxation optimized spectroscopy (TROSY) effect to facilitate NMR studies of nucleic acids larger than the median 30 nt size. Furthermore, we showcase the power of combining chemical and enzymatic synthesis to produce tailor‐made nucleic acid building blocks. Finally, we highlight the recently introduced palindrome‐nicking‐dependent amplification (PaNDA) to produce SI‐labeled single‐stranded DNAs^[^ ^16^ ^]^ and segmental labeling and site‐specific modifications by template‐directed tension (SegModTeX) technologies,^[^ ^17^ ^]^ which could dramatically transform the production of large site‐specific SI‐modified DNAs and RNAs with yields compatible for NMR spectroscopy.

## 2 Recent Advances in Stable Isotope Labeling of Nucleic Acids NMR

### 2.1 Stable Isotope Labeling of DNA for NMR

**Figure 1:**
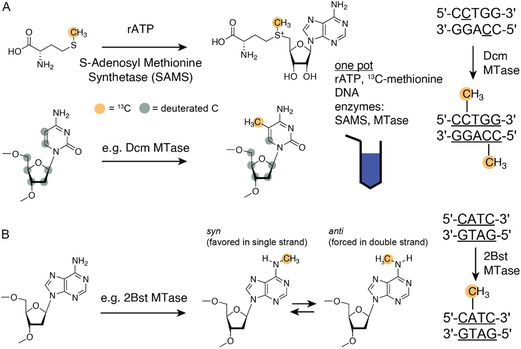
Methylation of DNA to unlock the ^13^C‐methyl TROSY effect.^[^ ^18^ ^]^ A) Methylation of C5 in 2′‐deoxycytidine in a one‐pot reaction. ^13^C‐methyl *S*‐adenosylmethionine is produced from ^13^C‐methyl methionine, rATP, and SAMS. In the same reaction vessel, the methylation of the 2′‐deoxycytidine at position C5 is catalyzed by an MTase. B) Methylation of N(6) in 2′‐deoxyadenosine (dA) in a one‐pot reaction. The equilibrium between *syn*‐ and *anti*‐conformation in m^6^dA is shown.

Kay and coworkers recently made the methyl TROSY experiment feasible for NMR studies of DNA in high‐molecular‐weight protein complexes.^[^ ^18^ ^]^ They added ^13^CH_3_ groups to specific DNA sites based on a one‐pot reaction in which addition of ^13^CH_3_‐methionine, *S‐*adenosylmethionine synthetase (EC 2.5.1.6) (SAMS), adenosine 5′‐triphosphate (ATP), the DNA of interest, and the required methyltransferase (MTase) is sufficient to catalyze these site‐specific methylations. For example, they achieved 5‐^13^C‐methyl‐deoxycytidine labeling with the *Escherichia coli* Dcm MTase that recognizes the sequence 5′‐C(A/T)GG‐3′ and catalyzes methylation of the C5 position of the cytosine closest to the 3′ end on each strand of the DNA to m^5^dC (**Figure** **1**A).

Alternatively, the methylation of the N(6) of 2′‐ dA was achieved by using MTases such as 2Bst or 1Bst (Figure 1B **)**. The ^13^C methyl group labeling was then used to probe the thermodynamics and kinetics of DNA melting in a 12mer duplex. The thermodynamics of the duplex melting was determined via peak volumes and the kinetics was determined by acquiring a magnetization exchange experiment. Further, for m^6^dA, the switching between the *syn*‐ and *anti*‐ conformation in the single‐stranded state was confirmed by a relaxation dispersion experiment reminiscent of m^6^A in RNA.^[^ ^19^ ^]^

Impressively, they were able to methylate a 153‐base pair (bp) Widom 601 DNA in the one‐pot mixture with four MTases: Msp (m^5^dC), 2Bst (m^6^dA), 1Bst (m^6^dA), and 2Mnl (m^6^dA). The main advantage of in vitro methylation procedure over in vivo approaches is that different methyl groups can be installed simply by using different enzymes without suppressing endogenous MTases, whose labels are not the desired methyl‐labeling pattern. A crucial consideration in getting high sensitivity TROSY spectra of m^5^dC is the deuteration of such large DNAs. Kay and coworkers showed that DNA deuteration leads to a ≈13‐fold signal to noise (S/N) ratio increase for m^5^dC in the 153‐bp Widom DNA. The m^5^dC methyl group embedded in a B‐form helix has a relatively high proton density, and thus, nearby proton nuclei lead to fast transverse relaxation. For the slowest relaxing methyl component in m^5^dC deuteration leads to a decrease of the R_2_ by a factor of 2–3. This contrasts with m^6^dA, where the methyl group has a relatively low proton density in a B‐form helix. Thus, deuteration has a rather negligible effect on the *R*_2_ and only a 1.2‐fold S/N ratio increase is observed between the ^2^H‐ and ^1^H‐DNA samples. Another contributing factor to the S/N enhancements between the deuterated and protiated 601 DNA is the longitudinal relaxation rate (*R*_1_). For m^5^dC, the *R*_1_ is twofold enhanced by deuteration, whereas, for m^6^dA, the *R*_1_ rate is further slowed down and thus the S/N ratio does not improve. The 200 kDa nucleosome core particle (NCP) is a crucial building block of chromatin and consists of four types of histone (H2A, H2B, H3, and H4) with two copies of each. A 150 bp DNA wraps around this octameric protein complex. Kay and coworkers used the DNA ^13^C methyl‐labeling approach to address the structure and dynamics of DNA in the free and histone‐bound state. The Widom 601 DNA—a sequence known for optimal wrapping around the NCP—was produced in quantities suitable for NMR by using a high‐copy‐number plasmid in either growth rich medium or in a minimal medium in deuterated water. Then, the isolated DNA was methylated as described above with four MTases: for m^5^dC Msp and for m^6^dA, 2Bst, 1Bst, and 2Mn1 were used. With DNA methyl labeling, the focus on the DNA was now possible with similar NMR TROSY studies performed on the isotopic labeled methyl groups in the side chains of isoleucine, leucine, and valine (ILV) amino acids in proteins in the NCP complex. The DNA methyl groups are well separated from resonances of ILV methyl labeling and a heteronuclear multiple quantum coherence  spectrum on a 150 μM NCP complex sample could be acquired in 40 min. The labeling strategy was used to derive order parameters for both protein and DNA methyl groups and only small variations of the S^2^_axis_ parameters were observed with the buried Msp methyl groups being the most rigid ones, while the least buried 2Bst and 1Bst show more flexibility as these methyl groups reside closer to the DNA termini. The authors also methylated CpG islands in the 601 DNA and then used ^13^C methyl groups of an ILV‐H2B sample to address structural changes of the protein component upon DNA methylation. The methylation of the nucleic acid component had a very small effect on the overall conformation of the H2B histone component and relaxation studies provided evidence that no change in the pico‐ to nanosecond dynamics of the protein occurs upon DNA methylation. These findings are in‐line with previous X‐Ray studies.^[^ ^20^ ^]^

### 2.2 Stable Isotope Labeling of DNA via the PaNDA Approach

**Figure 2:**
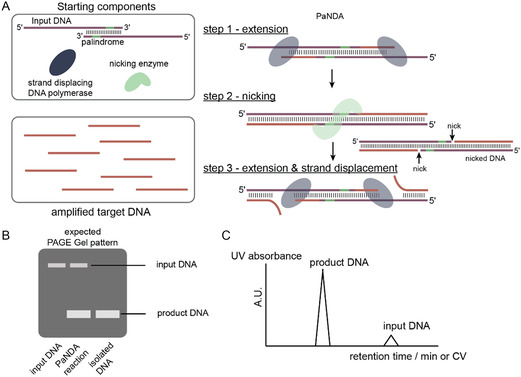
The PaNDA approach for SI labeling of single‐stranded DNA.^[^ ^16^ ^]^ A) The PaNDA procedure. B) Expected pattern of denaturing PAGE. C) Purification via anion exchange high‐performance liquid chromatography is facile due to the large charge difference between input and target DNA.

Increasingly, NMR spectroscopy is being harnessed to shed light on the structural and functional diversity of SI‐labeled DNA.^[^ ^15^ ^]^ One prime example is a detailed study of the RNA cleaving 10–23 DNA enzyme by the Etzkorn group.^[^ ^21^ ^]^ SI labeling played an important role in the study using ^13^C/^15^N and ^19^F labeling.^[^ ^22^ ^]^ The solid‐phase synthesis of DNA is a well‐established technique and was also used for residue‐specific SI labeling of various DNAs with the main applications in DNA quadruplex folding^[^ ^23^ ^]^ or the characterization of functional dynamics in DNA.^[^ ^24^ ^]^ Bottlenecks in the labeling procedure via solid‐phase synthesis include the high costs of the commercially available ^13^C/^15^N‐labeled DNA phosphoramidites or the laborious production of the SI‐labeled DNA building blocks for chemical synthesis, respectively. Alternatively, enzymatic methods for DNA SI labeling exist but are not as disseminated as the T7 RNA polymerase‐assisted SI labeling of RNA. A possible reason is the difficulty in separating the labeled DNA from the complementary DNA strand as they have the same concentration. Very recently, Iwahara and coworkers introduced the PaNDA technique to produce SI‐labeled single‐stranded DNAs, which solves some of the issues with the existing enzymatic methods.^[^ ^16^ ^]^ The approach requires three components—1) a strand displacing DNA polymerase, 2) a nicking enzyme, and 3) the input DNA. The input DNA functions as the template and the primer. This oligonucleotide comprises a replication template sequence followed by a palindromic primer partition. In the PaNDA approach, the polymerase extends this DNA sequence and produces double‐stranded DNA, which is further processed by the nicking enzyme leading to the cleavage at the start of the palindromic sequence. Thus, the target DNA is formed and the original DNA‐primer‐template is restored, which allows the strand displacing polymerase to extend the DNA template repeatedly (**Figure** **2**A). After the amplification step, during which even multiple doses of dNTPs can be added, the target DNA is easily isolated via anion exchange chromatography relying on the large charge difference between the input and target DNA (Figure 2B,C).

The PaNDA approach was used to produce various SI‐labeled single‐stranded DNAs, such as a 22 nt telomere repeat G‐quadruplex, the 26 nt therapeutic DNA aptamer NU172, and the 33 nt DNAzyme Dz5C. For the telomere repeat DNA, ^15^N isotope labeling was used to confirm the existence of multiple G‐quadruplex structures in the presence of potassium chloride. The NU172 aptamer inhibits thrombin in human blood and was used in phase II clinical trials for anticoagulation. The labeling was used to confirm the existence of a G‐quadruplex structure in the NU172 apo state. Further, the PaNDA approach was applied to characterize the half‐life of NU172 in human serum at 37 °C. Finally, the DNA enzyme Dz5C, extensively studied by Etzkorn and coworkers,^[^ ^21^ ^]^ was produced with ^13^C/^15^N dNTPs via the PaNDA method. This DNA, which proved to be catalytically active, gave high quality ^1^ H‐^13^C‐HSQC spectra in the substrate‐free and substrate‐bound state.

In conclusion, Iwahara and coworkers introduced an effective novel approach to produce SI‐labeled DNA, which will very likely be adopted by the nucleic acid NMR community, as the approach is highly economical and the enzyme components are commercially available.

### 2.3 Stable Isotope Labeling of RNA for NMR

**Figure 3:**
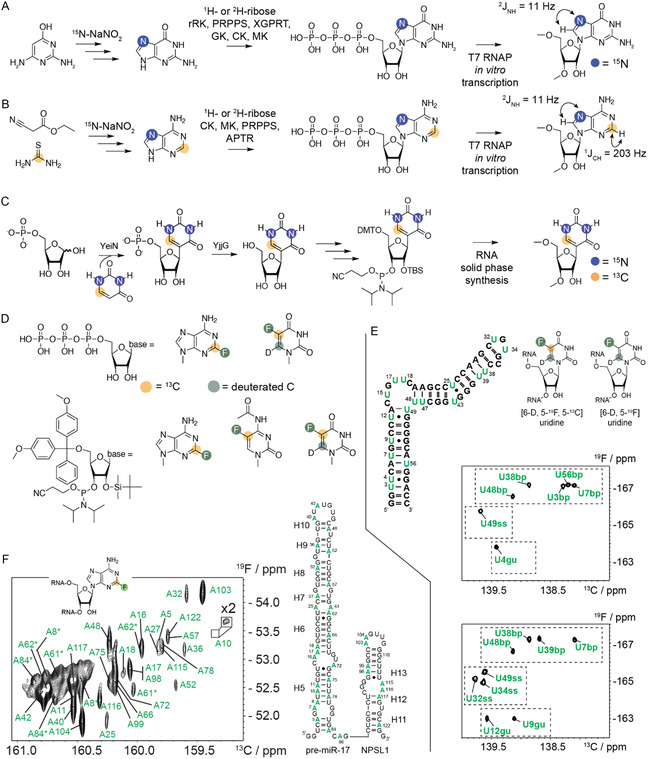
^15^N(7) purine,^15^N/^13^C pseudouridine, and ^19^F–^13^C‐labeling of aromatic nucleobases in RNA.^[^ ^25^, ^26^, ^29^, ^30^, ^36^ ^]^ Chemoenzymatic routes to A) ^15^N^7^‐rGTP, B) ^13^C(2)‐^15^N(7)‐rATP, and C) ^13^C/^15^N‐labeled pseudouridine phosphoramidite. D) Currently available RNA building blocks with aromatic ^19^F–^13^C‐labeling: 2‐^19^F‐2‐^13^C‐rATP, 5‐^19^F‐5‐^13^C‐6‐D‐rUTP and 2‐^19^F‐2‐^13^C‐adenosine phosphoramidite, 5‐^19^F‐5‐^13^C‐cytidine amidite, and 5‐^19^F‐5‐^13^C‐6‐D‐uridine amidite. E) Example of combinatorial labeling via solid‐phase synthesis site‐specific labeling to facilitate resonance assignment of 5‐^19^F‐5‐^13^C‐6‐D‐U modified RNA. The 5‐FU resonance further reflects the folding state (bp – base paired, ss – single stranded, gu – G•U bp). F) 2‐^19^F‐^13^C‐A labeling of a 124 nt oncomiR‐1 RNA with resonance assignment via a divide and conquer and a combinatorial labeling approach.

Bp‐specific labeling in RNA allows researchers to directly monitor Watson–Crick bps and the Hoogsteen face of nucleotides within large functional RNAs with complex folding. Optimized procedures have been proposed to synthesize chemoenzymatically either ^15^N(7)‐rGTP and ^13^C2‐^15^N(7)‐rATP or ^15^N(7)‐labeled purine phosphoramidite by using [7‐^15^N]‐adenine or [7‐^15^N]‐guanine (**Figure** **3**A,B).^[^ ^25^ ^]^ The former labeled bases are coupled to various labeled or unlabeled ribose to form the nucleoside triphosphate in a one‐pot reaction using enzymes from the salvage biosynthetic pathway. These nitrogen labeling schemes leverage the potential narrow linewidths of the ^15^N nuclei and make them applicable to large RNAs. Additionally, the N(7) atom, being in the major groove, serves as an atomic probe of binding events between nucleic acids and various ligands, such as metal ions, small molecules, or other hydrogen bond donors from nucleic acids or protein side chains.

By using a similar synthesis logic, recently a chemoenzymatic synthesis of isotopically labeled pseudouridine (Ψ) phosphoramidite was realized (Figure 3C).^[^ ^26^ ^]^ This is an important advance given the ubiquity of this post transcriptional modification—Ψ is found in transfer RNA (tRNA), ribosomal RNA (rRNA), and messenger RNA (mRNA) and a methylated derivative, N(1)‐methylpseudouridine (m^1^Ψ), is used to produce mRNA‐based vaccines. In vaccines, substituting U for m^1^Ψ makes the mRNAs less immunogenic and translated more efficiently in vivo.^[^ ^27^ ^]^ In fact, the m^1^Ψ modification in the mRNA‐based COVID‐19 vaccines is essential for their therapeutic efficacy. This efficient chemoenzymatic synthesis of ^13^C/^15^N‐labeled pseudouridine phosphoramidites enabled solid‐phase chemical synthesis of Ψ‐modified RNAs up to 75 nucleotides (nts), such as tRNA. The SI‐labeled pseudouridine 5′‐monophosphate (ΨMP) was produced enzymatically starting with chemically synthesized ^13^C/^15^N uracil and *YeiN*, a Ψ‐5′‐monophosphate *C*‐glycosidase in very high yield on the gram scale. Pyrimidine 5′‐monophosphate phosphatase *YjjG* removes the 5′‐phosphate group to yield the desired pseudouridine nucleoside (Ψ) with an overall isolated yield of ≈80 % and transformation of Ψ‐nucleoside into phosphoramidite proceeded with a yield of ≈50%. The availability of these SI‐labeled Ψ building blocks opens the door for analysis of Ψ‐modified nucleic acids by various biophysical tools, such as mass spectrometry as well as solution and solid‐state NMR spectroscopy.

To overcome the limitations of RNA based on the “traditional” SI labels of hydrogen‐1 (^1^H), phosphorus‐31 (^31^P), carbon‐13 (^13^C), hydrogen‐2 (^2^H), and nitrogen‐15 (^15^N), several researchers have turned to the magnetically active fluorine‐19 (^19^F) isotope to study RNA and protein structure and conformational changes, which occur upon ligand binding. ^19^F has favorable spectroscopic properties that make it an apt nucleus: it has high NMR sensitivity comparable to ^1^H (0.94 of ^1^H), 100% natural abundance, wider chemical shift dispersion than ^1^H and highly sensitive to changes in its local chemical environment, and is almost always absent in biological systems. This renders ^19^F a background‐free NMR probe. Drawing inspiration from the research of Arthanari, Wagner and coworkers^[^ ^28^ ^]^ on aromatic moieties of proteins and a 16‐nt DNA, we incorporated a ^19^F–^13^C spin pair into RNA nucleobases with improved spectroscopic features (Figure 3C). The uracil base (U) was readily synthesized from unlabeled potassium cyanide, [2‐^13^C]‐bromoacetic acid (label at C5), and ^15^N‐labeled urea (labels N(1)and N(3)). The resulting U was converted to 5‐fluorouracil (5‐FU) by direct fluorination with the electrophilic Selectfluor reagent.^[^ ^29^, ^30^ ^]^ For the chemoenzymatic approach, unwanted scalar coupling interactions were removed by selectively deuterating H6 (≈95%). Then, 5FU was coupled to *D*‐ribose labeled at the C1′ position to give 5F‐uridine triphosphate (UTP) using enzymes from the pentose phosphate and salvage pathways.^[^ ^29^ ^]^ This site‐specifically labeled 5 F‐UTP was readily accepted by phage T7 RNA polymerase to make RNA. For the solid‐phase synthesis method, the 5 F‐U was converted to the nucleoside using silyl‐Hilbert–Johnson nucleosidation reaction, position 6 is deuterated, and the 2′‐*O*‐tert‐butyldimethyl silyl group is then regioselectively introduced. The protected [5‐^19^F, 5‐^13^C, 6‐^2^H]‐uridine 3′‐*O*‐phosphoramidite obtained was readily transformed into [5‐^19^F, 5‐^13^C]‐cytidine 3′‐*O*‐phosphoramidite. The synthetic access to [5‐^19^F, 5‐^13^C]‐uridine (U) and ‐cytidine (C) phosphoramidites enabled the labeling of four model RNAs (HIV‐TAR 2 RNA, hHBV ε RNA, the 49 nt S‐adenosylmethionine (SAM) VI riboswitch aptamer from *Bifidobacterium angulatum* in the absence and presence of Mg^2+^ ions and SAM ligand, and the 59 nt oncogenic pre‐microRNA [miRNA]) with ^13^C/^19^F by solid‐phase RNA synthesis. Kreutz and coworkers adapted an economical and time‐saving strategy originally developed for DNA^[^ ^31^ ^]^ to assign all the ^19^F‐substituted uridine and cytidine resonances in all four RNAs. The number of sequences (*n*) determines the number of assignable resonances according to the equation 2^n^‐1, such that with five RNA sequences, 31 resonances can be assigned. To make the approach economical, they used a 25% label (i.e., 25% fold dilution of 100% [6‐^2^H, 5‐^19^F, 5‐^13^C]‐U labeled phosphoramidite with ^13^C‐unlabeled [6‐^2^H, 5‐^19^F]‐U phosphoramidite) to reduce the amount of the ^13^C/^19^F‐amidite needed for chemical synthesis. This translates to 26.1% for ^13^C such that the ^13^C isotopes increase by a factor of 23.7 compared to the natural abundance value of 1.1% for ^13^C. All other unlabeled uridine residues were replaced by [6‐^2^H, 5‐^19^F]‐Us to account for the nearest neighbor effect of ^19^F chemical shift perturbation.

### 2.4 Benefits of 5‐^19^F‐5‐^13^C‐U Labeling

Theoretical calculations predicted that, with increasing polarizing magnetic fields, the chemical shift anisotropy (CSA) mechanism will be the dominant contributor to the linewidth of the aromatic ^19^F–^13^C spin pairs.^[^ ^28^, ^29^ ^]^ Based on the calculated chemical shielding tensor for ^19^F–^13^C spin pairs using density functional theory, the computed TROSY R_2_ relaxation rates for the fluorinated carbons (^13^C_F_) of the ^19^F–^13^C pair of 5FU were estimated to be ≈2 times smaller than those of the protonated carbons (^13^C_H_) of the ^13^C–^1^H pair of U. In line with these simulations, NMR experiments indicated the ^13^C_F_ TROSY component relaxes ≈2 times slower than the ^13^C_H_ TROSY component in a 30‐nt (≈10 kDa) human immunodeficiency type 2 transactivation response (HIV‐2 TAR) RNA and the 61‐nt (≈20 kDa) human hepatitis B virus encapsidation signal epsilon (hHBV *ε*) RNA elements. Thus, the mere expediency of placing a ^13^C spin next to a ^19^F label slows down the fast relaxation of the ^13^C nuclei within a ^19^F–^13^C spin pair. In addition to this favorable ^13^C_F_ TROSY linewidth, ^19^F–^13^C labeling confers ≈6‐fold improvement in chemical shift dispersion of ^19^F compared with ^1^H and comparable dispersion in ^13^C. The RNAs investigated show a chemical shift dispersion of 2.6–4.5 parts per million (ppm) in the ^19^F dimension and only 0.5–0.8 ppm in the ^1^H dimension. Intriguingly, replacing ^1^H with ^19^F reduces chemical shift dispersion along the ^13^C dimension from 2.1–2.3 to 1.5–1.7 ppm. Overall, these results suggest the slight diminished loss in dispersion along the ^13^C dimension is more than offset by the marked gain in resolution in the ^19^F dimension afforded by the ^19^F–^13^C spin pair in 5‐FU RNAs compared with the ^1^H–^13^C spin pair. In addition to these spectacular gains in resolution and favorable linewidths, the high sensitivity of the ^19^F nucleus enables us to unambiguously delineate helical Watson–Crick base paired, nonhelical as well as GU wobble regions (Figure 3D). Based on the chemical shift distributions alone, the ^19^F–^13^C correlations of HIV‐2 TAR and hHBV ε could be correlated as follows. Nonhelical Us resonate around ≈−165.5 ppm in ^19^F and ≈142.5 ppm in ^13^C, the Us in helical regions are centered around ≈−167.5 ppm in ^19^F and ≈141.5 pm in ^13^C as reported earlier,^[^ ^32^ ^]^ and finally, the G•U wobble signals resonate at ≈−163.5 ppm in ^19^F and ≈142 pm in ^13^C, again consistent with earlier observations of G•U bps in tRNA.^[^ ^33^ ^]^ With ^1^H–^13^C spectra, helical residues can be distinguished from nonhelical residues. Yet, nonhelical residues cannot be differentiated from G•U bps. This distinguishing feature, though not available for ^1^H–^13^C spin pairs, is now possible with the high sensitivity of ^19^F within a ^19^F–^13^C spin pair. Given the increased resolution, favorable linewidths, and ^19^F chemical shifts as sensitive markers of a nucleotide's folding state, the [5‐^19^F, 5‐^13^C] labels are suitable for addressing structural dynamics and the detection of ligand binding to RNA. This is particularly significant for the discovery of small drug‐like molecules that act as inhibitors. Like proteins, RNAs contain specific binding pockets that can be targeted with small molecules.^[^ ^34^ ^]^ As an example, by labeling the single cytidine C26 with ^19^F–^13^C of the SAM VI aptamer, the ligand binding event could be monitored by observing the imino proton region of ^1^H‐NMR spectra followed by ^13^C‐decoupled ^19^F NMR spectra, which revealed two conformations in the apo state: a major state (95%) signal at −166.8 ppm and a minor state (5%) at −165.4 ppm. This minor state has the chemical shift signature of a bound‐like conformational state with a closed G9–C26 bp. Ligand binding reshuffles this minor population to adopt a novel major resonance (75%) at −165.0 ppm. These results underscore a recurrent theme that riboswitch conformational selection is a key feature in the ligand recognition process such that a 5% ligand binding competent state of the free RNA active nucleates the SAM ligand docking in response to external cues, such as ions, ligands, pH, temperature, or other cellular signals.^[^ ^35^ ^]^ As a second example, titrating the small molecule raloxifene into 5‐FU hHBV ε RNA leads to specific chemical shift perturbations (CSPs) of a subset of the nonhelical region within the 6‐nt bulge but not anywhere else in the RNA. Minor CSPs are observed from U residues flanking the 6‐nt bulge, embedded in the helical portion of the 5‐FU hHBV RNA, as well as a U within a G•U flanking the proposed binding pocket.^[^ ^29^ ^]^

### 2.5 Extension to 2‐^19^F‐2‐^13^C‐A and Advantages Over 5‐^19^F‐5‐^13^C‐U

As predicted and experimentally verified, these ^13^C–^19^F‐labeled C and U nucleotides exhibited favorable spectroscopic properties of ≈6‐fold ^19^F chemical shift dispersion and ^13^C linewidths of the ^19^F–^13^C spin pairs that are approximately twice as sharp as the corresponding ^1^H–^13^C spin pairs in NMR TROSY experiments.

Nonetheless, theoretical simulations using the Spinach software package predicted that [2‐^19^F, 2‐^13^C] adenosine spin pair should outperform the ^13^C–^19^F labeled pyrimidine nucleotides by a factor of three in their linewidths.^[^ ^28^ ^]^ Recently, the production of [2‐^19^F, 2,8‐^13^C_2_] adenosine RNA phosphoramidite with two flavors of [2‐^19^F, 2‐^13^C] adenosine RNA triphosphates was reported: one with a regular triphosphate functionality and a nonhydrolysable analog with a CH_2_‐bridge between P_γ_ and P_β_.^[^ ^36^ ^]^ The phosphoramidite was made starting with labeled hypoxanthine, turned into inosine, converted via a chlorination step into adenosine, benzoylation and a selective nitration, followed by a substitution reaction using tetrabutylammonium fluoride as the economical ^19^F source. This intermediate gives the desired 2‐^19^F‐^13^C‐key intermediate, which after standard sugar protection and functionalization steps yields the desired RNA phosphoramidite. In a new approach, the [2‐^19^F, 2‐^13^C] adenosine ribose triphosphates are made starting from a cheap commercially available unlabeled adenosine. The oxidation of the unlabeled adenosine, whose ribose sugars are protected, gives the N1 oxide that undergoes a Dimroth rearrangement^[^ ^37^ ^]^ with ^13^C potassium cyanide as an economical ^13^C source for C2. The fluorine atom at position 2 is introduced using a regioselective Balz–Schiemann reaction with hydrogen fluoride serving as the ^19^F‐source to replace the 2‐amino group.^[^ ^38^ ^]^ Finally, chemical triphosphorylation reactions give the requisite 2‐^19^F‐^13^C‐ATP and the nonhydrolysable ATP analog.

As alluded to earlier, theoretical calculations predict that the maximum TROSY effect for the 2‐FA ^13^C_F_ component has a 3‐ to 5‐fold improvement in linewidth compared to the CH components. With these labels in hand, RNAs with sizes ranging from 60 to 124 nts with [2‐^19^F, 2‐^13^C]‐adenosine labels were synthesized. As predicted, the [2‐^19^F, 2‐^13^C] spin pair has a maximum fivefold TROSY gain over the [2‐^1^H, 2‐^13^C] spin pair, with a ^13^C_F_ TROSY resonance linewidths of ≈2.6–3.1 Hz compared to ^13^C_H_ TROSY linewidths of ≈8.5–12.9 Hz. This contrasts with uridine 5‐^13^C_F_ TROSY resonance linewidths of 8.5–13 Hz, which are comparable to 2‐^13^C_H_ adenosine TROSY resonances. Indeed, at 25 °C, direct measurements of the R_2_ for the ^13^C_F_ TROSY component of the 61 nt hHBV RNA give values of 10–12.5 s^−1^, and at 0 °C, the rates increase to 33–40 s^−1^, a factor of 3–4. Unlike the ≈6‐fold increase in resolution observed with 5‐FU, the chemical shift dispersion for ^19^F (2.5 ppm) in the carbon fluorine (CF) correlation spectrum is only ≈1.5× that of ^1^H in the C_H_ spectrum. However, compared to the ≈0.3× observed with 5‐FU, the C_F_ (1.8 ppm) carbon dispersions are ≈0.5× that of C_H_ (3.3 ppm). Hence, it is clear that despite the reduced carbon chemical shift dispersion, the ^19^F‐^13^C‐A labeling approach outperforms the 5‐^19^F‐5‐^13^C‐pyrimidine methodology with regard to very sharp linewidths, and therefore, should be the method of choice for RNAs larger than 100 nt. This novel labeling method allowed us to probe the structural dynamics of a large 124 nt oncomiR‐1, an RNA encoding 6 microRNAs (miRs).^[^ ^39^ ^]^ As these miRs are implicated in the development of cancer, they are of great biological and medical interest. Here, the triphosphate building block is critical to label such a large RNA with 2‐^19^F‐^13^C‐A. Solid‐phase synthesis becomes inefficient for such large RNAs. For instance, based on the E^N^ rule, a coupling efficiency of E = 0.98 gives a yield of 8.2% for an RNA of length N = 124. Given the large size and overlap, to assign almost all resonances, a divide and conquer/combinatorial labeling approach cross‐validated with site‐directed compensatory (i.e., replace the A—U bps with G=C bps in Watson–Crick‐based regions) mutagenesis was proposed, after making sure that mutagenesis does not significantly perturb the structure of the target RNA nor impairs function (Figure 3E). For bulge and loop residues, it is important to minimize the formation of alternative folds, guided by secondary structure prediction algorithms (e.g., MCsym pipeline) for sequence selection. Two other useful applications of the novel CF labeling include a ^13^C detected ^19^F Carr–Purcell–Meiboom–Gill relaxation dispersion experiment and NMR probe of the phosphate donor function in kinase‐catalyzed reactions. For the larger RNAs, the ^13^C–^19^F labeling is expected to resolve the ^19^F resonance overlap seen in the 1D ^19^F spectrum.

The nonhydrolysable 2‐^19^F‐^13^C‐labeled ATP analog allows for assessing the role of ATP binding in large kinases or kinase–protein signaling complexes. In binding of our analog to nucleoside monophosphate kinase, a 10‐fold line broadening of the kinase‐bound ^19^F resonance relative to the apo state was observed. In the ^19^F‐^13^C‐TROSY experiment, instead only a doubling of the ^13^C linewidth was reported. Thus, the 2‐^19^F‐^13^C‐spin pair in the ATP analogs have the potential to address the role of ATP in large kinases or protein complexes that use ATP as a cofactor by solution, solid‐state, and in‐cell NMR.

### 2.6 Limitations of 2‐^19^F‐2‐^13^C‐A and 5‐^19^F‐5‐^13^C‐U

Substitution of ^1^H with ^19^F in uridine and adenosine nucleotides of RNA has extensively been shown to be largely nonperturbing, in the context of structural and thermodynamic properties.^[^ ^40^, ^41^ ^]^ Furthermore, this modification has been shown to be potentially nonperturbing at the functional level, as most fluorinated RNAs still retain binding properties comparable with those of the wild type. Despite these studies, incorporation of ^19^F into RNAs is still not completely innocuous, as it significantly alters the magnetic properties of proximal and distal atoms, which can readily be detected by NMR.^[^ ^29^, ^32^, ^36^, ^40^, ^41^, ^42^ ^]^

Cytidine triphosphate synthetase (CTPS) converts UTP to CTP. However, upon fluorination of UTP to 5F‐UTP, CTPS is unable to achieve this conversion, hinting at potential subtle structural changes, which the enzyme is efficient at discriminating.^[^ ^29^ ^]^ Another possible explanation for the ablation of CTPS activity in 5F‐UTP is the introduction of significant polarization changes within the aromatic ring of the nucleobases upon ^19^F incorporation.^[^ ^41^ ^]^ Thus, some potential electrostatic interactions essential for enzyme recognition at the active site may be altered. Changes in the electrostatic polarity directly impact chemical shift resonances of neighboring nuclei, since the net external magnetic field a nucleus experiences at its resonance frequency is dependent on the distribution of electrons around that nucleus. As such, chemical shift resonances for RNAs made with 2F‐ATP and 5F‐UTP modifications need to be reassigned, relative to the wild‐type RNA. The changes in chemical shift resonances seem to be more pronounced in 2F‐ATP‐labeled RNAs.^[^ ^29^, ^36^ ^]^ Although some chemical shift assignment strategies were discussed earlier, assignment becomes particularly challenging as the RNA increases in size, necessitating the need for synthesis of RNA constructs with various labeling patterns for reassignment purposes. The sharpening of linewidths by the TROSY effect in 2F‐ATP and 5F‐UTP for the ^13^C–^19^F spin pair is only experienced by the ^13^C_F_ nucleus. Therefore, the narrow linewidths of these isotopic labels cannot be leveraged when directly observing ^19^F signals. In fact, very broad lines are observed for ^19^F in large RNAs and at high magnetic field strength, owing to its large CSA. Take for instance, the acquisition of a ^1^H–^1^H nuclear overhauser effect spectroscopy (NOESY) experiment, which is indispensable in structure calculation and refinement by providing distance restraints. Although the ^1^H density increases exponentially in larger RNAs, leading to line broadening and signal overlap, the ^13^C/^15^N in conjunction with deuteration have been used as editing tools to make ^1^H–^1^H NOESY amenable to very large RNAs.^[^ ^43^ ^]^ While experiments, such as ^1^H–^19^F heteronuclear overhauser spectroscopy (HOESY) and ^19^F–^19^F NOESY, exist to probe these spatial interactions in the context of ^19^F, their applications are normally limited to small and midsized RNAs. ^1^H–^19^F HOESY NMR cannot be run on any generic NMR probe, as it requires ^1^H and ^19^F nuclei in different channels for decoupling. Additionally, in very large RNAs with large correlation times, a classic ^13^C–^19^F TROSY pulse program, where polarization starts and is detected on ^19^F (out‐and‐back design) may not only lead to broader signals but also reduced sensitivity due to the increased ^19^F_C_ transverse relaxation rates in larger RNAs. Hence, fully leveraging TROSY benefits of the ^13^C–^19^F spin pair requires special probes that permit ^13^C‐ and ^19^F‐detected out‐and‐stay or ^19^F‐ and ^13^C‐detected out‐and‐stay TROSY experimental designs.^[^ ^28^ ^]^

## 3 Approaches to Segmental and Site‐Specific Labeled RNA

**Figure 4:**
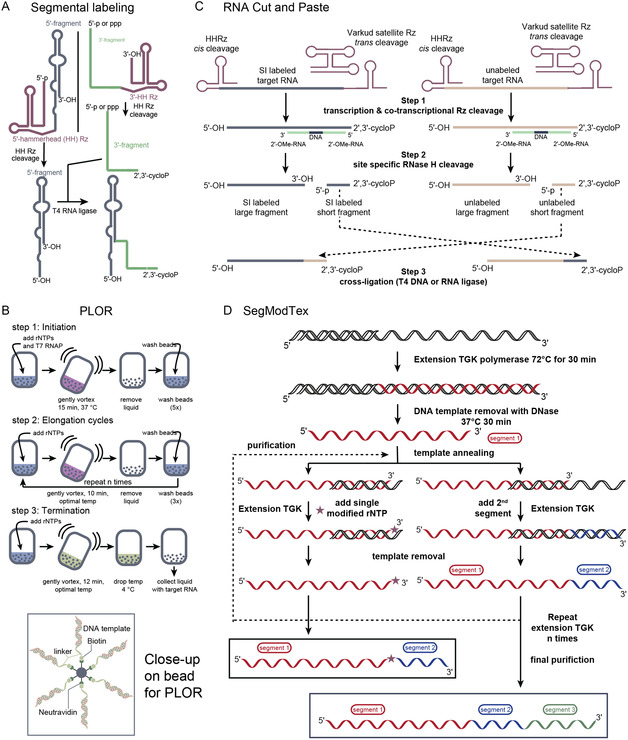
Approaches for segmental labeling of RNA for NMR studies. A) Segmental RNA labeling for NMR as proposed by Kim, Lukavsky, and Puglisi.^[^ ^15^ ^]^ B) PLOR approach by Wang and coworkers.^[^ ^43^ ^]^ C) Cut and Paste approach introduced by Duss and Allain.^[^ ^44^ ^]^ D) SegModTex methodology as described by D’Souza and coworkers^[^ ^17^ ^]^ and Hocek and coworkers.^[^ ^48^ ^]^

Kim, Lukavsky, and Puglisi established the segmental labeling approach about 22 years ago on a 100 kD hepatitis C virus internal ribosome entry site IRES RNA. In this approach two transcripts, one SI‐labeled and the other unlabeled and thus NMR silent, are stitched together by the T4 RNA ligase. The first transcript is 5′ flanked by a ribozyme to give an RNA with a 5′‐ and 3′‐hydroxyl. The second transcript is primed with guanosine monophosphate in the in vitro transcription reaction and outflanked with a 3′‐ribozyme to produce a 2′,3′‐cyclophosphate, which avoids self‐ligation and makes the ligation of the two transcripts possible (**Figure** **4**A).^[^ ^15^ ^]^

In 2015, the position‐selective labeling of RNA (PLOR) method was introduced.^[^ ^44^ ^]^ The approach uses a streptavidin bead to which the biotin‐tagged DNA template binds. Then, several rounds of T7 RNA polymerase transcription are initiated and stopped, governed by the rNTP composition. Here, SI‐labeled rNTPs can be used to selectively label residues in certain RNA stretches. The method gave highly satisfying results for selected RNAs (e.g., adenine riboswitch) but the general applicability of the approach remains unrealized (Figure 4B).

In another approach, Allain and coworkers introduced a simple modular RNA cut and paste approach to generate 1) small isotopically labeled RNAs for NMR structural studies, 2) large segmentally isotope and/or spin‐labeled RNAs for diamagnetic NMR and paramagnetic relaxation enhancement NMR, and 3) large spin‐labeled RNAs for pulse EPR spectroscopy (Figure 4C).^[^ ^45^ ^]^

Very recently, D’Souza and coworkers introduced a very elegant alternative to established methods—the SegModTeX technique (Figure 4D).^[^ ^17^ ^]^ This technique is very promising and uses the mutant TGK DNA polymerase, which comprises the classic steric‐gate mutation in the active site (Y409G)^[^ ^46^ ^]^ and a second‐gate mutation (E664K) in the thumb subdomain, to be a RNA polymerase with a high acceptance for modified ribonucleotides triphosphates.^[^ ^47^ ^]^ In their work D’Souza and coworkers demonstrated that a large variety of rNTPs can be incorporated with the TGK polymerase and that several segments can be added to an RNA, giving easy access to segmentally labeled RNAs with sizes up to a few hundred nucleotides. In a very similar approach, Hocek and coworkers used again the engineered thermophilic DNA polymerase TGK but also the SFM4‐3 for the expedient production of site‐specific nucleobase‐labeled or hypermodified RNA, albeit at much reduced concentrations.^[^ ^48^ ^]^

Noteworthy, the SegModTex approach was used to introduce 2‐F‐adenosine in target RNAs paving the way for residue‐specific 2‐F‐2‐^13^C‐A labeling in large RNAs, and thereby, harnessing the very favorable TROSY effect in the aromatic ^19^F‐^13^C‐spin topologies described in the previous section.

**Figure 5:**
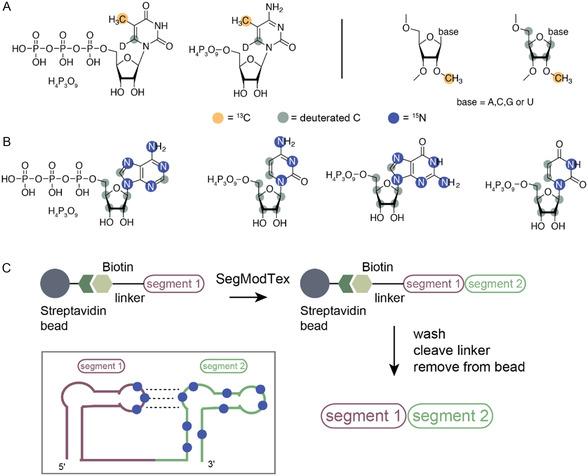
SI labeling schemes for large RNAs and the potential introduction of the labels via a revised SegModTex approach. A) Proposed ^13^C‐methyl‐labeled nucleotides for large RNAs. B) Perdeuterated ^15^N‐labeled RNA triphosphates for the elucidation of secondary and tertiary structure of large ribonucleic acids by NMR. C) SegModTex V2 approach–segment 1 obtained from solid‐phase RNA synthesis with a 5′‐linker and biotin handle is attached to a streptavidin bead. Then, the SegModTex procedure is carried out as described before. Finally, the target RNA is washed, the linker is cleaved, and the desired segmentally labeled RNA is obtained. This approach could be useful to probe tertiary structure interactions in larger constructs as shown schematically.

Further the approach is not only amenable to introducing naturally occurring modified RNA residues (m^6^A, Ψ, s^2^U, and m^5^C), fluorescence labels but also nucleotides giving the possibility for post synthetic labeling (5‐Aminoallyl‐C/U or 5‐ethynyl‐U). This has considerably expanded the toolbox of BioNMR for GRNA. For example, paramagnetic tagging of large RNA becomes feasible with the SegModTex approach together with the readout of the paramagnetic relaxation enhancements (PRE) effect by selected SI‐labeled residues.^[^ ^49^ ^]^ In addition, PRE‐tagged RNAs will become useful in studying large ribonucleoprotein complexes, in which the RNA radical tags induce the PRE effect in the protein complex. In a similar fashion, 1,4,7,10‐tetraaza‐cyclododecane‐tetraacetic acid (DOTA)‐based lanthanide chelating tags leading to long‐range structure information via pseudocontact shifts will be of high importance in such systems.^[^ ^50^ ^]^ In parallel, efficient synthetic routes to rNTPs with SI labeling patterns suitable for higher molecular weight systems will be needed. We think that deuteration in combination with atom‐specific ^13^C/^15^N labeling will be a key feature of such rNTPs. For example, 5‐^13^CH_3_‐uridine and cytidine triphosphates with a deuterium in position 6 are highly promising candidates as effective reporter nuclei in larger RNAs, very similar to methylated residues in DNA as described by Kay and coworkers (see previous section, **Figure** **5**A). Alternatively, the ^13^C methyl group could be integrated as a 2′‐*O*‐CH_3_‐nucleotide, a widespread naturally occurring modification. The approach was recently successfully used to address RNA annealing kinetics within a cell cycle associated protein 1‐induced phase condensate, where the high viscosity leads to very unfavorable relaxation properties.^[^ ^51^ ^]^ The work was mainly motivated by successes in the protein NMR field with using methyl groups as reporters of both structure and dynamics in high‐molecular‐weight systems. A single 2′‐*O*‐methyl group minimally perturbs the RNA's thermodynamics (stabilization <0.3 kcal mol^−1^), and further, the resonance positions of such a 2′‐*O*‐^13^CH_3_ group (≈3.5 ppm ^1^H and ≈60 ppm ^13^C chemical shift) are isolated from (natural abundance) peaks of the protein scaffold. Ideally, the 2′‐*O*‐^13^CH_3_ group should be embedded within a perdeuterated sugar environment to eliminate effective relaxation partners, such as the H1′, H2′, H3′, and H4′ protons (Figure 5A **).**

To address secondary and tertiary structures of larger RNAs, atom specific ^15^N‐labeled deuterated rNTPs will be effective labels (Figure 5B). Together with the SegModTex approach, certain sequence stretches can be probed for long‐range interactions by introducing ^15^N labels (Figure 5C). Using SegModTex‐placed ^15^N labels, scientists can now shed light on the structural features of larger RNAs, for example, riboswitch aptamers with the adjacent expression platform; they can also address equilibrium dynamics in such systems by using NMR experiments, such as the chemical exchange saturation transfer method.

We further foresee potential improvements in the SegModTex approach by transferring the methodology to the solid phase. This was recently realized by immobilizing the DNA template.^[^ ^52^ ^]^ The approach could be further simplified by immobilizing the first RNA segment. Segment 1 could be produced via RNA solid‐phase synthesis with a photocleavable linker group and a biotin handle^[^ ^53^ ^]^ to attach the first segment to a streptavidin bead (Figure 5C). Furthermore, residue‐specific SI labeling can be introduced at this stage with ^13^C and ^15^N labeled RNA phosphoramidites. The bead‐bound segment 1 can then be elongated by the SegModTex method and subsequent washing, template removal, and elongation steps will be significantly facilitated as compared to the original approach. The final product RNA can then be liberated from solid support via an orthogonal release reaction. We anticipate that this approach could be useful to probe tertiary structure interactions in larger RNAs as shown (Figure 5C).

## 4 Conclusion and Outlook

### 4.1 DNA SI Labeling

The recent advances in SI labeling of DNA for NMR studies represent significant progress in understanding the structure and dynamics of DNA, particularly in the context of large molecular complexes, such as nucleosomes and DNA–protein interactions. The methyl TROSY experiment pioneered by Kay and colleagues offers a powerful tool for investigating DNA dynamics in high‐molecular‐weight complexes. This methodology has shown great promise in elucidating DNA conformational changes, DNA–protein interactions, and the thermodynamics and kinetics of DNA melting processes, all of which are crucial for a deeper understanding of cellular processes like gene regulation and chromatin remodeling. The development of the PaNDA approach by Iwahara and coworkers offers an attractive alternative to traditional solid‐phase synthesis for SI labeling of DNA. This enzymatic method addresses several bottlenecks in DNA labeling, such as high cost and intensive labor, making it a more accessible and efficient technique for research groups. These advancements in SI labeling techniques will likely have a transformative impact on the study of DNA at the atomic level. As the NMR community increasingly adopts these methodologies, we can expect further refinement in experimental techniques and the exploration of more complex DNA systems.

### 4.2 RNA SI Labeling

The novel chemoenzymatic synthesis of isotopically labeled nucleotide analogs has enabled the synthesis of modified RNAs of substantial size, which is vital for investigating biologically significant RNAs such as riboswitches, microRNAs, and those involved in viral replication. The incorporation of ^19^F–^13^C spin pairs in pyrimidine and purine nucleotides has further expanded the possibilities for high‐resolution studies of large RNAs. The improved spectroscopic properties of ^19^F–^13^C labeling, especially of 2‐F‐^13^C‐A, include sharper linewidths and enhanced chemical shift dispersion. These properties are particularly advantageous for the study of large RNAs, where traditional methods often struggle with resolution and sensitivity. Looking forward, the field of RNA structural biology will benefit from continued innovations in isotope labeling. The ability to label larger RNA constructs with high precision will enable more detailed investigations into the dynamics and functional mechanisms of complex RNAs involved in a variety of biological processes, including gene regulation, RNA splicing, and translation. Furthermore, the integration of these advanced labeling strategies with other biophysical techniques, such as mass spectrometry and cryo‐EM, will provide a more comprehensive picture of RNA function.

### 4.3 Approaches to Segmental and Site‐Specific Labeled RNA

The segmental labeling of RNA has undergone significant advancements over the past two decades, offering powerful tools for the structural and dynamic analysis of large, complex RNA molecules. The SegModTex technique has proven to be a promising advance, as it allows for the incorporation of a wide variety of modified nucleotides and enables the construction of segmentally labeled RNAs of considerable size. The flexibility of this approach, coupled with its ability to integrate both SI and site‐specific modifications, has opened new possibilities for the study of RNA dynamics in more complex environments. The future of RNA structural and dynamic studies using segmental labeling approaches appears highly promising. Continued refinement, along with the development of efficient synthetic routes for isotope‐labeled rNTPs, will likely enable the synthesis of even larger RNAs with tailored modifications. Such advancements will be crucial for the study of RNAs involved in essential biological processes, including riboswitches, long noncoding RNAs, and RNA–protein complexes.

## Conflict of Interest

Christoph Kreutz is an advisor to and holds an ownership interest in Innotope, a company providing RNA SI‐labelling products. The remaining authors declare no competing interests.
